# Immune Response after Anti-SARS-CoV-2 mRNA Vaccination in Relation to Cellular Immunity, Vitamin D and Comorbidities in Hemodialysis Patients

**DOI:** 10.3390/microorganisms12050861

**Published:** 2024-04-25

**Authors:** Egle Dalinkeviciene, Brigita Gradauskiene, Sandra Sakalauskaite, Kristina Petruliene, Ruta Vaiciuniene, Inga Skarupskiene, Daina Bastyte, Jolanta Sauseriene, Leonas Valius, Inga Arune Bumblyte, Edita Ziginskiene

**Affiliations:** 1 Department of Nephrology, Lithuanian University of Health Sciences, LT-50161 Kaunas, Lithuania; kristina.petruliene@lsmu.lt (K.P.); ruta.vaiciuniene@lsmu.lt (R.V.); inga.skarupskiene@lsmu.lt (I.S.); ingaarune.bumblyte@lsmu.lt (I.A.B.); edita.ziginskiene@lsmu.lt (E.Z.); 2 Department of Immunology and Allergology, Lithuanian University of Health Sciences, LT-50161 Kaunas, Lithuania; brigita.gradauskiene@lsmu.lt; 3 Laboratory of Immunology, Department of Immunology and Allergology, Lithuanian University of Health Sciences, LT-50161 Kaunas, Lithuania; sandra.sakalauskaite@lsmu.lt (S.S.); daina.bastyte@lsmu.lt (D.B.); 4 Department of Family Medicine, Lithuanian University of Health Sciences, LT-50161 Kaunas, Lithuania; jolanta.sauseriene@lsmu.lt (J.S.); leonas.valius@lsmu.lt (L.V.)

**Keywords:** COVID-19, anti-SARS-CoV-2, vaccination, hemodialysis, immune response, T cells, vitamin D

## Abstract

In the global threat of SARS-CoV-2, individuals undergoing maintenance dialysis represent a vulnerable population with an increased risk of severe COVID-19 outcomes. Therefore, immunization against SARS-CoV-2 is an essential component of healthcare strategy for these patients. Existing data indicate that they tend to exhibit a reduced immune response to vaccines compared to the general population. Our study aimed to assess both humoral and cellular immune responses following two doses of an anti-SARS-CoV-2 mRNA vaccine, an ability to maintain adequate antibody titers over time, and potential relations with vitamin D, comorbidities and other factors in hemodialysis patients based on a single center experience. A total of 41/45 patients (91.1%) responded to the second dose of the anti-SARS-CoV-2 mRNA vaccine. The titer of anti-SARS-CoV-2 IgG class antibodies and levels of T cells three to four weeks after vaccination were lower in dialysis patients than in healthy controls. Antibodies titer in dialysis patients had a positive correlation with B lymphocytes and was related to cardiovascular diseases. The level of CD4+ cells had a negative correlation with hemodialysis vintage, as did the vitamin D level with post-vaccination seroconversion and decline in anti-SARS-CoV-2 antibodies titer during six months after vaccination. Hemodialysis patients had decreased amounts of CD4+ and CD8+ cells and lower levels of anti-SARS-CoV-2 antibodies than healthy controls. Therefore, chronic hemodialysis could lead to diminished cellular immunity and humoral immune response to the anti-SARS-CoV-2 mRNA vaccination and reduced protection from COVID-19. Comorbidity in cardiovascular diseases was associated with a lower level of specific anti-SARS-CoV-2 antibody titer. Vitamin D may be important in maintaining stable levels of anti-SARS-CoV-2 antibodies, while the duration of dialysis treatment could be one of the factors decreasing anti-SARS-CoV-2 antibody titer and determining lower CD4+ cell counts.

## 1. Introduction

Some groups of people, such as the elderly, patients with chronic diseases and immunosuppressed, are particularly vulnerable and have a higher risk of severe COVID-19 disease and associated mortality [1]. Another group of patients who have an increased risk of infection and death compared to the general population are patients on kidney replacement therapy [2]. Data from clinical studies show that end-stage kidney disease (ESKD) patients have a weaker immune response to vaccination than the general population due to immune dysregulation [3]. A lower immune response to hepatitis B vaccine in dialysis patients is identified in clinical trials [4,5]. There are data indicating that the cellular and humoral immune responses after vaccination with an mRNA-based vaccine induced in hemodialysis patients are lower compared to healthy controls, and they are at high risk of reinfection due to compromised immunity [6]. When evaluating hemodialysis patients, it was found that the overall antibody response after full vaccination was 89%, and a faster decline in anti-SARS-CoV-2 antibody titers was observed compared to the general population [7,8]. Antibody response is generally thought to ensure protection against initial infection, and the induction of virus-specific neutralizing antibodies in the airways is regarded as the most probable predictor of future protection following natural infection or vaccination [9]. However, the cellular immune response recognizes and controls intracellular pathogens and represents an essential mechanism for limiting viral infections, which should also be crucial to the response against COVID-19 infection [10].

The reasons why patients on kidney replacement therapy have a weaker innate and adaptive immune system responsible for the immune response are multifaceted. They include—uremia-induced suppression of the immune system, which leads to a reduced cell-mediated and antibody-mediated immune response and a faster decrease in antibody titers due to impaired functions of T and B lymphocytes [11]. In addition, other risk factors have been identified, such as older age, diabetes, obesity, cancer, cirrhosis, cardiovascular disease, malnutrition and inflammation, oxidative stress, which impair the protective immunity of hemodialysis patients [12]. Some authors suggest that lower serum albumin, higher doses of intravenous iron sucrose, insufficient vitamin D, and erythropoietin supplementation may also contribute to a weaker immune response [13]. Consequently, vitamin D deficiency observed in ESKD patients might contribute to a diminished anti-inflammatory and increased pro-inflammatory status. Such a state of chronic inflammation might impair the generation of pathogen-specific immunity [14]. Previous studies evaluating the cellular and humoral responses to vaccines have shown that humoral response is lower and delayed in hemodialysis subjects compared to control individuals. However, the researchers emphasize that further studies are necessary to more comprehensively evaluate and understand the effectiveness of SARS-CoV-2 vaccination in hemodialysis patients [6,7].

Vaccines against SARS-CoV-2 are relatively new. Despite the increasing number of studies on efficiency, there is currently a lack of data on the cellular immune response of hemodialysis patients to mRNA vaccines because many studies are more focused on the humoral response after vaccination against COVID-19. In addition, controversial data can be found [6]. There are insufficient data on vitamin D’s effect on the immune response in dialysis patients. Therefore, additional knowledge of the effectiveness and factors determining the immune response in hemodialysis patients is still highly relevant. Our study aimed to assess both humoral and cellular immune response following two doses of an anti-SARS-CoV-2 mRNA vaccine, an ability to maintain adequate antibody titers over time, and potential relations with vitamin D, comorbidities and other factors in ESKD patients undergoing dialysis.

## 2. Materials and Methods

The prospective study was carried out to evaluate the response to vaccination against SARS-CoV-2 in the Hospital of Lithuanian University for Health Sciences Kauno klinikos, which is the largest dialysis center in the Baltic States, with extensive experience in the field of kidney replacement therapy, and provides high-quality patient care.

The study comprised two cohorts: one consisted of patients with ESKD undergoing maintenance hemodialysis (referred to as the dialysis group), and the other consisted of healthcare workers without known diseases that may influence study results (referred to as the healthy control group) affiliated with our institution. The approval of The Regional Bioethical Committee was obtained on 5 February 2021 (No. BE-2-43). Forty-five individuals undergoing maintenance hemodialysis for over three months and 48 healthy control subjects without chronic kidney disease were included in the study. All study subjects received two doses of BNT162b2 (Pfizer-BioNTech) anti-SARS-CoV-2 mRNA vaccine, adhering to the manufacturer’s recommended 21-day interval between doses. The vaccination was carried out during the second wave of the pandemic. All study subjects received the first dose of the vaccine between 27 December 2020 and 12 January 2021: healthcare workers in the period of 27 December 2020–5 January 2021, hemodialysis patients—during 6 and 12 January 2021). All patients and healthy control subjects agreed to participate in the study and provided signed informed consent. Patients receiving immunosuppressive drugs, previously transplanted, were excluded from the study. Groups were matched by age. None of these individuals had a history of COVID-19 disease, and their COVID-19 PCR tests before, during, and after vaccination were negative. A real-time PCR analysis method was applied to detect SARS-CoV-2 RNA using a GeneProof SARS-CoV-2 PCR Kit (Brno, Czech Republic). Blood samples were collected to assess anti-SARS-CoV-2 antibodies and lymphocyte subpopulations three to four weeks after the second dose of vaccine for subjects of both groups. Their demographic data and information about comorbid conditions were gathered from their medical records. According to the medical documentation, the controls did not have chronic kidney disease.

A more detailed analysis was carried out in patients undergoing hemodialysis. Information about the presence of diabetes, malignancy and cardiovascular diseases was collected in dialysis patients. None of the patients had active oncological disease or were being actively treated for malignancy. Angina pectoris, previous myocardial infarction, or stroke were considered as cardiovascular diseases. Patients did not take vitamin D supplements during the study.

Immunoglobulins (Ig)—G, M, A were tested simultaneously as the titer of anti-SARS-CoV-2 antibodies and levels of lymphocyte subpopulations. Data about vascular access, dialysis vintage (the period in months from the start of hemodialysis treatment till study) of dialysis patients were collected from their medical documentation. Levels of creatinine, hemoglobin, calcium, phosphorus, albumin, C-reactive protein, parathyroid hormone, 25-hydroxyvitamin D, and dialysis dose according to spKt/V [15] were performed as routine tests according to standard practice before and after vaccination. Blood samples for evaluation of anti-SARS-CoV-2 antibodies in dialysis patients were taken one more time six months after vaccination (n = 39), and the change in anti-SARS-CoV-2 antibodies titer between three to four weeks and six months after the second dose of vaccine was evaluated (Δ anti-SARS-CoV-2 titer). Patients were followed for COVID-19 disease after vaccination until July 2023.

### 2.1. Measurement of Anti-SARS-CoV-2 Antibodies

For quantitative in vitro determination of human antibodies of the IgG class against SARS-CoV-2 spike proteins in serum, QuantiVac ELISA assay (Euroimmun, Lübeck, Germany) was applied. The values of the research results were given in BAU/mL (BAU—binding antibody units). According to the manufacturer’s instructions, a result ≥35.2 BAU/mL was interpreted as seropositive, and subjects were classified as vaccine “responders” and “non-responders”.

### 2.2. Assessment of Lymphocyte Subpopulations

The flow cytometry (BD FACSLyric™, BD Biosciences, San Diego, CA, USA) technique was used for quantification of lymphocyte subpopulations in blood. First, blood incubation procedures with monoclonal antibodies mixes (BD Multitest™ 6-color TBNK reagent, BD Biosciences, San Diego, CA, USA) and erythrocytes lysis were performed. The prepared samples were analyzed on the BD FACSLyric system with BD FACSuite Clinical software v1.2.1 (BD Biosciences, San Diego, CA, USA). The lymphocyte region was gated, and the absolute numbers (cells/L) of lymphocyte subpopulations in the sample were determined. T, B and natural killer cells were characterized by CD3+, CD4+, CD8+, CD19+ and CD16+/56+ expression.

### 2.3. Evaluation of Vitamin D

Serum 25-hydroxyvitamin D levels were evaluated in the cold season. The assessment of the concentration of 25-hydroxyvitamin D in serum was determined by the enzyme-linked immunosorbent assay ELISA using DI Asource 25OH vitamin D Total ELISA kit (Louvain-la Neuve, Belgium). The analysis kit detection limit was defined as the apparent concentration two standard deviations below the average OD at zero binding, namely 1.5 ng/mL.

### 2.4. Statistical Methods

The software package SPSS 29.0 was used for data storage and statistical analysis. Continuous variables were expressed as mean and standard deviation (SD) or median and interquartile range (Q1–Q3), as appropriate. Frequency tables (numbers and percentages) were used for categorical variables. For non-parametric continuous variables, the Wilcoxon Signed-Ranks Test was used. Quantitative data distribution was evaluated using the Kolmogorov–Smirnov test. Spearman’s correlation coefficient was used to determine the correlation between variables (cases of non-normal distribution were observed). To compare the quantitative sizes of two independent samples when the distribution of variables was normal, the Student’s *t*-test was used. In contrast, non-normally distributed variables were analyzed using the Mann–Whitney U test, and in multiple group comparisons—the Kruskal–Wallis test. Using chi-square (χ^2^) criteria, the interdependence of qualitative evidence was evaluated. The McNemar test was used to analyze paired nominal data. Univariate binary logistic regression analysis was performed to evaluate the importance of dialysis vintage to CD4+ level, and multivariate binary logistic regression analysis was performed to evaluate the importance of dialysis vintage and vitamin D level to the decrease in anti-SARS-CoV-2 titer (Δ anti-SARS-CoV-2 titer) within six months after anti-SARS-CoV-2 mRNA vaccination in the dialysis patients group. The results were considered statistically significant when *p* < 0.05 for all analyses.

## 3. Results

The baseline characteristics of dialysis patients are presented in Table 1. The mean age of healthy controls was 64.3 ± 12.0 years, and there was no difference in age between the study groups (*p* = 0.139). The gender distribution was 13 (27.1%):35 (72.9%) (male/female) in healthy control group (*p* < 0.001 compared to dialysis patients).

A total of 41/45 patients of the dialysis group (91.1%) responded (anti-SARS-CoV-2 ≥ 35.2 BAU/mL) to the second dose of the anti-SARS-CoV-2 mRNA vaccine. Meanwhile, all subjects in the healthy control group had anti-SARS-CoV-2 titer ≥ 35.2 BAU/mL.

The mean titer of anti-SARS-CoV-2 antibodies IgG class was 1008.6 ± 1005.5 vs. 2464.7 ± 1771.1 (*p* < 0.001) three to four weeks after the second vaccine dose in hemodialysis patients and individuals of the healthy control group, respectively.

The results from cellular immunity markers showed that levels of T lymphocytes (CD3+), cytotoxic T cells (CD8+), T helper cells (CD4+), B lymphocytes (CD19+), and natural killer cells (CD16+/56+) were higher in the healthy control group than in dialysis patients. All results are given in Table 2.

**Figure 1:**
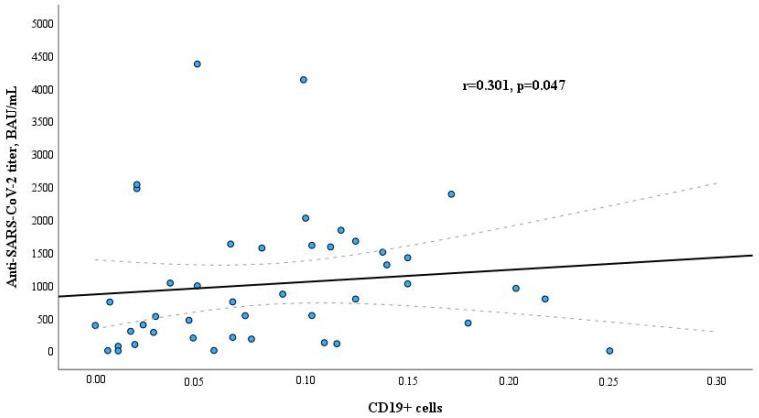
Relation between CD19+ cells and anti-SARS-CoV-2 titer in the blood of dialysis patients.

**Figure 2:**
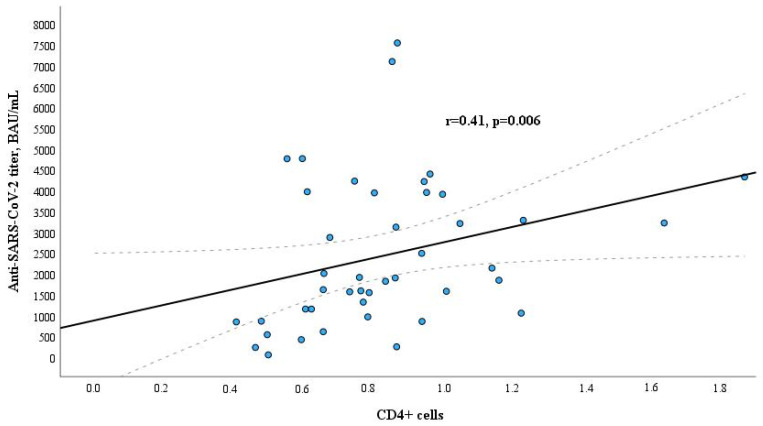
Relation between CD4+ cells and anti-SARS-CoV-2 titer in the blood of the healthy control group.

When evaluating the relation between levels of lymphocyte subpopulations and anti-SARS-CoV-2 antibodies IgG titer, different results were obtained in the study groups. Only B lymphocytes (CD19+) had a weak positive significant correlation with anti-SARS-CoV-2 antibodies IgG titer in the dialysis patients group (r = 0.301, *p* = 0.047, Figure 1); association of levels of other lymphocyte subpopulations was found not statistically significant. In contrast to the dialysis group, a positive significant correlation between T helpers (CD4+) and anti-SARS-CoV-2 antibodies IgG titer was found in the healthy control group (r = 0.41, *p* = 0.006, Figure 2). There were no other statistically significant associations between lymphocyte subpopulations and anti-SARS-CoV-2 antibodies IgG titer in this group. The relation between CD19+ cells and anti-SARS-CoV-2 titer in the blood of healthy controls is presented in Figure S1, and the relation between CD4+ cells and anti-SARS-CoV-2 titer in the blood of dialysis patients is in Figure S2.

After evaluating the impact of demographic factors on the formation of anti-SARS-CoV-2 antibodies, it was determined that older subjects had a greater response to anti-SARS-CoV-2 mRNA vaccination in the healthy control group: a statistically significant positive correlation was found between the titer of anti-SARS-CoV-2 antibodies and age (r = 0.440, *p* = 0.003). Nevertheless, in the dialysis group, there was no significant correlation between these two factors; only a tendency to inverse correlation was observed (r = −0.283, *p* = 0.06).

There was no statistically significant relation between anti-SARS-CoV-2 antibody titer and sex in the healthy control group (*p* = 0.134) and in the dialysis patients after randomly selecting an appropriate number of men (*p* = 0.082).

There were no statistically significant associations between levels of T helper cells (CD4+), cytotoxic T cells (CD8+), and age (*p* = 0.087 and *p* = 0.117, *p* = 0.844 and *p* = 0.143) or sex (*p* = 0.396 and *p* = 0.347, *p* = 0.292 and *p* = 0.372) in both study groups (dialysis patients and healthy controls, respectively).

When assessing the influence of comorbidities on the immune response after two doses of anti-SARS-CoV-2 mRNA vaccine in dialysis patients, the results showed no significant relation between diabetes, malignancy and antibody production, levels of CD4+, CD8+ cells. Cardiovascular diseases were associated with the humoral immune response: anti-SARS-CoV-2 antibodies titer in dialysis patients with concomitant cardiovascular diseases was lower if compared with patients without this comorbidity (830.89 ± 936.06 BAU/mL vs. 1445.91 ± 1073.55 BAU/mL, respectively). There was no statistically significant difference between levels of T helpers (CD4+) and cytotoxic T cells (CD8+) and the presence of cardiovascular diseases. The findings are presented in Table 3.

**Figure 3:**
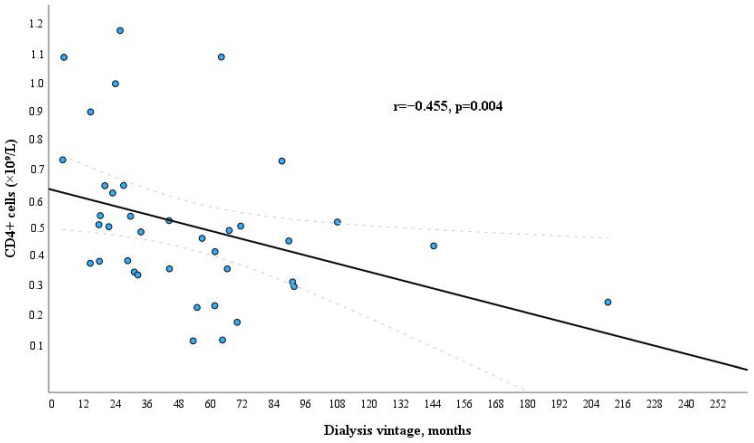
Relation between dialysis vintage and level of CD4+ cells in the dialysis patients group.

Analysis of clinical characteristics of dialysis patients showed no statistically significant relations between immune response, as assessed by the level of anti-SARS-CoV-2 antibodies titer, T helper cells (CD4+), cytotoxic T cells (CD8+) and type of dialysis vascular access, serum levels of hemoglobin, albumin, C-reactive protein, calcium, phosphorus, parathormone, IgG, IgM, IgA, spKt/V, ESKD etiology. Only 25-OH vitamin D levels before vaccination had a significant relationship with a titer of anti-SARS-CoV-2 antibodies, and hemodialysis vintage in months with a level of T helper cells (CD4+) (negative correlations were obtained). Higher levels of 25-OH vitamin D in the blood led to weaker anti-SARS-CoV-2 antibody production. Longer duration of dialysis treatment resulted in lower levels of CD4+ cells (Figure 3). Only a tendency to inverse correlation was observed between dialysis vintage and levels of cytotoxic T cells (CD8+) (r = −0.307, *p* = 0.06). Univariate binary logistic regression analysis confirmed the relationship between hemodialysis vintage and levels of CD4+ cells. Since the sample variables were non-normally distributed, we used the median of the level of T helpers (CD4+) in the dialysis patients group (0.49 × 10^9^/L). The cohort was divided into two groups: those with lower levels of CD4+ cells after vaccination (<0.49 × 10^9^/L) and those with higher levels (≥0.49 × 10^9^/L). The results of univariate binary logistic regression analysis for evaluation of dialysis vintage as a factor relevant to lower level of CD4+ cells (<0.49 × 10^9^/L) after anti-SARS-CoV-2 mRNA vaccination are: Odds Ratio (95% CI)—4.829 (1.213–19.219), *p* = 0.022.

The results of the clinical characteristics analysis are detailed in Table 4.

**Figure 4:**
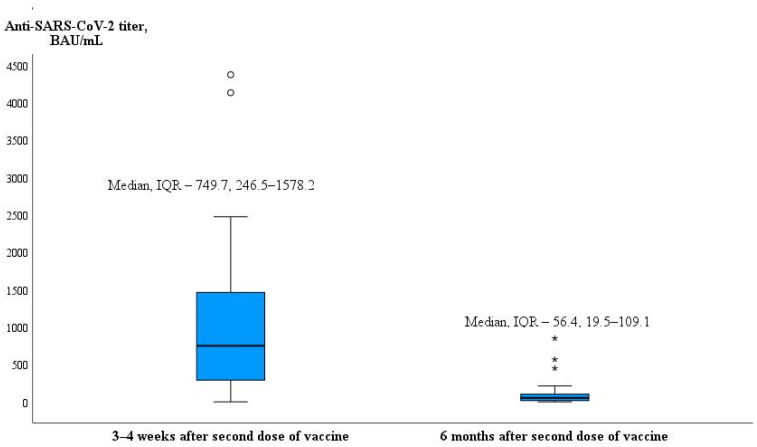
Box Plot of change in anti-SARS-CoV-2 titer during six months after vaccination in dialysis patients (minimum data value, lower quartile value, median value, upper quartile value, maximum data value, outliers).

In evaluating the ability to maintain adequate antibody titers over time, the findings indicated a significant reduction in the IgG titer of anti-SARS-CoV-2 antibodies among hemodialysis patients within six months following their second vaccine dose (1008.6 ± 1005.5 BAU/mL vs. 110.3 ± 167.3 BAU/mL and median [25–75%] 749.7 [246.5–1578.2] vs. 56.4 [19.5–109.1], respectively, *p* < 0.001) (Figure 4).

The percentage of vaccine non-responder dialysis patients increased from 8.9% to 35% during six months after anti-SARS-CoV-2 mRNA vaccination (*p* = 0.002, Figure S3).

**Figure 5:**
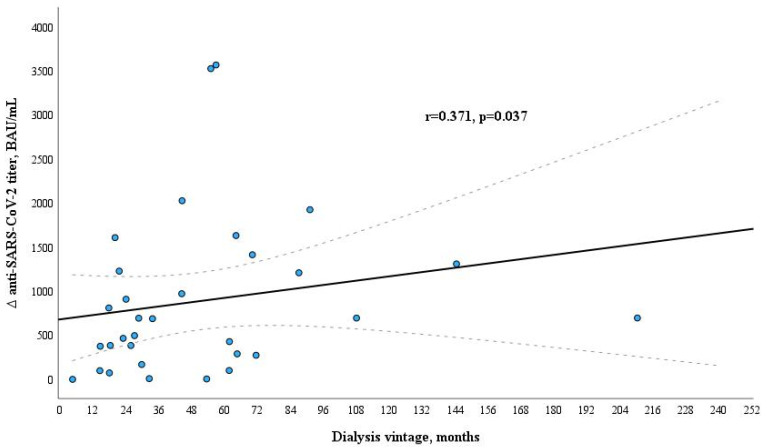
Relation between dialysis vintage and Δ anti-SARS-CoV-2 titer during six months after vaccination in the dialysis patients group.

In assessing the decline in anti-SARS-CoV-2 titer (Δ anti-SARS-CoV-2 titer) during six months after anti-SARS-CoV-2 mRNA vaccination, it was determined that longer dialysis vintage was associated with greater reduction in anti-SARS-CoV-2 antibodies IgG titer (Figure 5). However, the sustained immune response was stronger in dialysis patients with longer dialysis treatment (a positive correlation was found between the dialysis vintage in months and the anti-SARS-CoV-2 titer six months after vaccination (r = 0.415, *p* = 0.015).

**Figure 6:**
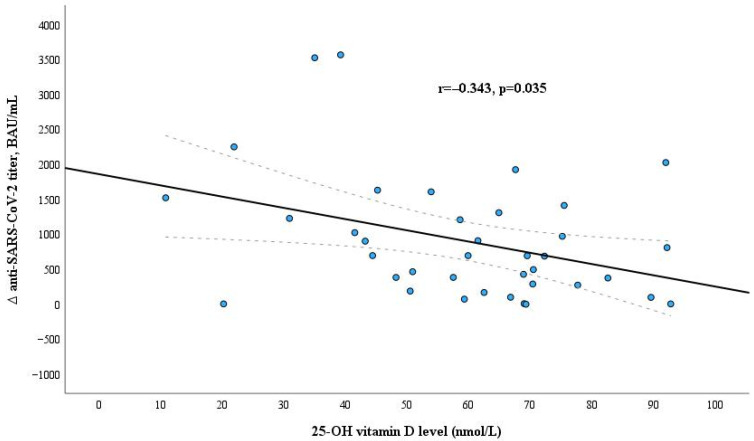
Relation between serum 25-OH vitamin D level and Δ anti-SARS-CoV-2 titer during six months after vaccination in the dialysis patients group.

The results of the six-month follow-up showed that higher vitamin D levels in the dialysis patient’s blood after vaccination were associated with lower anti-SARS-CoV-2 antibodies IgG titer decline during six months after vaccination (Figure 6).

Since the sample variables were non-normally distributed, we used the median of the Δ anti-SARS-CoV-2 titer within six months after anti-SARS-CoV-2 mRNA vaccination in the dialysis patients group (690 BAU/mL). The cohort was divided into two groups: those with lower decline in anti-SARS-CoV-2 titer during six months after vaccination (<690 BAU/mL) and those with higher decline in anti-SARS-CoV-2 titer during this period (>690 BAU/mL). Binary logistic multivariate regression analysis was performed to evaluate the importance of dialysis vintage and vitamin D levels in the decline in anti-SARS-CoV-2 titer (Δ anti-SARS-CoV-2 titer) during six months after anti-SARS-CoV-2 mRNA vaccination. Dialysis vintage and vitamin D level of dialysis patients remained significant factors to the decline in anti-SARS-CoV-2 titer. Comparison data of these groups and the results of binary logistic multivariate regression analysis are given in Table 5.

Out of all vaccinated patients in the dialysis group, 21 (46.7%) patients had COVID-19 disease (follow-up period until July 2023). The last case was observed in August 2022. Only one patient required hospitalization. The initial level of anti-SARS-CoV-2 IgG class antibodies titer after the second dose of vaccine did not differ between dialysis patients who subsequently developed and did not develop COVID-19 disease (896.39 ± 1030.2 BAU/mL vs. 1066.28 ± 1021.68 BAU/mL, respectively, *p* = 0.448).

## 4. Discussion

This research aimed to assess the immune response induced by the mRNA vaccine against SARS-CoV-2 in individuals undergoing maintenance hemodialysis. The findings are based on the results from a single center which happens to be the largest dialysis center in the Baltic States and has extensive experience in the field of kidney replacement therapy.

Our results revealed that the majority of patients undergoing maintenance hemodialysis developed a significant humoral response after receiving two doses of the vaccine. However, cellular immunity, as indicated by the number of CD4+ and CD8+ cells, was diminished in the dialysis patients group. Despite an adequate humoral response, dialysis patients showed a significantly lower response than the healthy control group. The threshold for a positive response in our assay was set at ≥35.2 BAU/mL, and a substantial majority (91.1%) of our hemodialysis patients exceeded this limit. This closely aligns with the response rate of 88.78% reported in a recently published systematic review, which encompassed 27 studies involving a total of 1337 hemodialysis patients [19], as well as in other studies [20,21].

Examining humoral immune responses after vaccination against COVID-19 is valuable, but it is essential to evaluate additional cellular immune responses, particularly in immunosuppressed individuals like hemodialysis patients. Those undergoing maintenance dialysis exhibit a diminished response to vaccination due to uremia-related immune system suppression, leading to disruptions in T lymphocytes and antigen-presenting cells [11,12]. Our study showed that levels of T lymphocytes (CD3+), cytotoxic T cells (CD8+), T helper cells (CD4+), B lymphocytes (CD19+) and natural killers cells (CD16+/56+) were higher in healthy controls than in the dialysis patients group. This is consistent with the results from the ROMANOV study [22]. This study highlighted the compromised immune response among hemodialysis patients without a previous COVID-19 infection, particularly in terms of cellular response. CD4+ T cells were detectable in only 50% of hemodialysis patients, as opposed to 100% in healthy volunteers, and CD8+ T cells were detectable in just 31% of hemodialysis patients compared to 70% in healthy volunteers. According to the Sanders J.S.F. study, SARS-CoV-2-specific T-cell responses after the second dose of vaccination were lower in patients on dialysis (52.6%) than in those in the general population (75.0%) [23].

In contrast to the dialysis group, the study revealed a significant positive correlation between T helper cells (CD4+) and anti-SARS-CoV-2 antibody IgG titers in the healthy control group (r = 0.41, *p* = 0.006). CD4+ cells play a crucial role in coordinating adaptive immune responses by their ability to recruit and offer assistance to various immune effectors while also engaging in direct effector functions [24,25,26]. We also showed that the amount of CD4+ T cells had a negative correlation with the dialysis vintage and did not correlate with the amount of anti-SARS-CoV-2 antibody titers. It suggests that the production of antigen-specific effector memory CD4+ T cells after vaccination, crucial to achieving an adequate humoral response, is impaired in dialysis patients.

In our study, only B lymphocytes (CD19+) had a positive significant correlation with anti-SARS-CoV-2 antibodies IgG titers in the maintenance dialysis patients group (r = 0.301, *p* = 0.047). The association with levels of other lymphocyte subpopulations was found not statistically significant. B lymphocytes play a pivotal role in the human defense against viral infections by generating targeted antibodies. Additionally, they are crucial for preventing infectious diseases through vaccination [27]. Therefore, it is no coincidence that a positive correlation between B lymphocytes (CD19+) and anti-SARS-CoV-2 antibodies IgG titers in the maintenance dialysis patients group was obtained. The same results were obtained in the study by Duni A. and co-authors. The authors assert that despite CD19+ B cell counts falling below normal reference values in hemodialysis patients, the positive correlation observed with antibody production affirms the triggering of the humoral immune response after BNT162b2 vaccination [28]. The dialysis group had a lower level of CD4+ and CD19+ cells in their blood compared with the control healthy group. This suggests that their immune response is diminished, which resulted in a worse vaccination response.

All individuals in the healthy control group had a seroconversion after the second dose of the anti-SARS-CoV-2 mRNA vaccine. The greater amount of B lymphocytes (CD19+) in dialysis patients led to a higher amount of anti-SARS-CoV-2 antibodies, while gender and age had no effect. The study showed that the number of anti-SARS-CoV-2 mRNA vaccine responders decreased from 91.1% to 65% during six months after vaccination, and higher vitamin D levels in blood serum of dialysis patients were negatively correlated while dialysis vintage was directly associated with the decrease in anti-SARS-CoV-2 antibodies levels during the mentioned period.

The existing data indicate that COVID-19 vaccines might exhibit reduced efficacy in populations with compromised immune systems, including patients with chronic kidney disease (CKD) [29,30]. In a systematic review that assessed the immunogenicity and efficacy of COVID-19 vaccines in various immunosuppressed populations, within the dialysis group, the percentage of non-responders varied from 2 to 30%, a range lower than that observed in other groups such as solid organ transplant recipients (18–100%) and patients with hematological malignancy (14–61%) [30]. The mean of anti-SARS-CoV-2 IgG class antibodies titers three to four weeks after the second vaccine dose was more than twice lower in hemodialysis patients as compared to controls in our study. Similarly, in other studies, patients undergoing dialysis exhibited lower antibody titers than the general population [20,21,31,32,33]. The results of the study from Israel were similar to ours. In this study, Yanay B. et al. reported anti-SARS-CoV-2 antibody levels 21 to 35 days after the second dose of the BNT162b2 COVID-19 mRNA vaccine. They found that the median anti-SARS-CoV-2 antibody levels were statistically significantly lower in dialysis patients compared to controls (116.5 [IQR 66–160] AU/mL vs. 176.5 [IQR, 142–235] AU/mL). However, more than 90% of the dialysis group exhibited seropositivity [34].

When examining the immune response in relation to other factors, we did not determine the correlation between the age of dialysis patients and anti-SARS-CoV-2 antibody titers and number of CD4+ and CD8+ cells. Similar to our findings, there was no statistically significant correlation between age, gender, or age at the beginning of hemodialysis treatment and antibody production in the Tsoutsoura P. study [11]. On the other hand, in the Grupper A. et al. study, there was a notable inverse relationship between advancing age and antibody levels in both examined groups. For every age category, the healthy control group displayed higher antibody levels than the dialysis group, a significant difference for ages below 60 and between 60 and 70 years old [31]. Jahn M. et al. also assessed the immunogenicity of the mRNA-based vaccine BNT162b2 in chronically ill patients. Consistent with our study findings, all hemodialysis patients showed antibody titers above the ELISA cutoff. However, these levels were significantly lower than those in a healthy control group of healthcare workers. Contrary to our results, higher age correlated with lower antibody titers (r^2^ = 0.2954, *p* < 0.0001) in hemodialysis patients [35]. According to our data in the dialysis group, there was no significant correlation between the antibody level and age, a tendency to negative correlation was observed (r = −0.283, *p* = 0.06). This result may have been due to a sample size that was too small.

We did not obtain any statistically significant correlation between the anti-SARS-CoV-2 antibody titers, levels of T helpers (CD4+), cytotoxic T cells (CD8+) of maintenance dialysis patients and dialysis dose (spKt/V), hemoglobin, albumin, C-reactive protein, calcium, phosphorus, parathormone blood level, CKD etiology, separately with diabetes, malignancy. Contrasting with our findings, Agur T. et al. showed that lower serum albumin and higher doses of intravenous iron were negative predictors of antibody response. Conversely, factors like younger age, serum albumin levels over 3.5 g/dL, lower intravenous iron doses, and a body mass index under 30 kg/m^2^ were identified as positive predictors for an antibody response [36]. With co-authors, Van Praet J. [20] and Anand S. [37] also found similar correlations between immune response and serum albumin levels. Premuzic V. et al., as well as our study, did not find associations between hemoglobin, serum calcium, phosphates, creatinine levels and immune response [33].

In our study, lower anti-SARS-CoV-2 antibody titer was associated with cardiovascular diseases in the hemodialysis patients group. Still, we did not obtain significant relations between cardiovascular diseases and markers of the cellular immune response. Contrary to our results, cardiovascular comorbidities were not associated with the humoral immune response to the anti-SARS-CoV-2 vaccine in other studies [38,39]. On the other hand, other authors, when evaluating the data on the immune response of anti-SARS-CoV-2 vaccines in dialysis patients, found that higher ESKD comorbidity index score, which includes cardiovascular diseases, along with other comorbidities, was associated with lower antibody response [40,41]. The value of cardiovascular disease in these findings remains unclear. Our study’s results confirm previous findings of Cozzolino M. [42] that evaluating the interrelationship between cardiovascular diseases and immune response in hemodialysis patients is very important, especially since cardiovascular diseases are present in >50% of dialysis patients. No data could be found in the literature on associations of cardiovascular diseases with T cells after anti-SARS-CoV-2 mRNA vaccination.

In research conducted by Broseta J. and colleagues involving 205 individuals undergoing dialysis who received either the mRNA-1273 or BNT162b2 vaccine, it was observed that 97.7% of the 175 initially seronegative patients developed a response (humoral, cellular, or both). Among these patients, 95.4% underwent seroconversion. Factors such as the use of immunosuppressive treatment, extended dialysis duration, lower hemoglobin and albumin levels, and reduced counts of white blood cells and lymphocytes were identified as statistically significant predictors of a lack of response in the univariate analysis. In the multivariable analysis, immunosuppressive treatment and lower albumin levels retained statistical significance [43]. More studies have found that longer dialysis vintage was associated with a higher risk for absent or attenuated response [33,37]. In our study, the total amount of anti-SARS-CoV-2 antibodies was not related to the dialysis vintage (in months). Similar results were shown in Lioulios G. et al.’s study. This study found no correlation between dialysis vintage and serum antibody levels against SARS-CoV-2 S1 protein or neutralizing antibody titers [44]. However, our study revealed a negative correlation of the amount of CD4+ T cells with the dialysis vintage. Thus, the duration of dialysis treatment is important for the developing cellular immunity. The results of previous studies [32,33,45,46] showed that the amount of anti-SARS-CoV-2 antibodies decreased rapidly a few months after vaccination in patients with kidney diseases. Our results are in line with these studies. The anti-SARS-CoV-2 antibody titers significantly decreased after six months compared to the anti-SARS-CoV-2 antibody IgG titers three to four weeks after vaccination, and the percentage of vaccine non-responders in maintenance dialysis patients increased from 8.9% to 35%. Furthermore, we determined that dialysis vintage (in months) and quantitative decline in specific anti-SARS-CoV-2 antibodies titer after vaccination during the time (between 3–4 weeks and 6 months) had a positive correlation in hemodialysis patients (r = 0.371, *p* = 0.037 and *p* = 0.021 according to multivariate binary logistic regression analysis). In contrast to our findings in the study by Jahn M. and colleagues, the duration of hemodialysis dependency showed no association with changes in antibody titers (r^2^ = 0.0007, *p* = 0.8261) [35]. Stumpf J. et al. discovered that a brief period on dialysis posed a risk factor for a significant decline in IgG levels following vaccination [47]. This was also linked to an elevated risk of seroconversion failure [29], a conclusion that contradicts the results reported by other researchers [33,37]. The authors suggest that the humoral immune system may adapt or recover during a more extended period of dialysis therapy in stable dialysis patients. This speculation could also elucidate our obtained results, indicating that although the anti-SARS-CoV-2 antibody titers decreased more rapidly, the sustained immune response six months after vaccination was more robust in dialysis patients with longer duration of dialysis treatment.

Vitamin D is recognized as an immune function regulator, influencing innate and adaptive immune responses [48]. Vitamin D directly impacts the function of monocytes, macrophages and dendritic cells (DCs) and the secretion of related cytokines [49]. The active form of 1,25-dihydroxyvitamin D has potent anti-inflammatory properties by switching a more inflammatory Th1/Th17 response to a less inflammatory—Th2/Treg response. As a result, the secretion of pro-inflammatory mediators (e.g., interferon-gamma (IFN-g), TNF-a, IL-1b, IL-6, IL-8, IL-12, IL-17) decreases, and the production of anti-inflammatory cytokines (IL-4, IL-10) increases [50]. It is shown that vitamin D status is associated not only with the vaccine against COVID-19 but also with other vaccine efficacy in CKD patients. Zitt E. and colleagues illustrated that CKD patients with serum vitamin D levels below 10 ng/mL exhibited a reduced seroconversion rate when administered the hepatitis B vaccine [51]. Vitamin D may also impact serological response against SARS-CoV-2 [52], but the role of the response to the anti-SARS-CoV-2 vaccine remains unclear. Furthermore, the obtained results are controversial. Some studies showed that higher vitamin D concentrations in blood serum were positively related to greater anti-SARS-CoV-2 antibody response in healthy adults. On the other hand, there are data about the fact that vitamin D was not associated with anti-SARS-CoV-2 seropositivity [53,54,55]. Nevertheless, there is no objection to the idea that vitamin D contributes to a better course of COVID-19 [56]. In contrast to these assertions, our study findings revealed that higher levels of anti-SARS-CoV-2 antibodies were linked to lower vitamin D levels in hemodialysis individuals. Studies [57,58] also identified that lower vitamin D levels were associated with greater immune response without statistical significance. In this case, we obtained statistically significant data. Incidentally, higher antibody titers were detected in patients with low vitamin D levels after vaccination against Human Papillomavirus [59]. Moreover, we determined that vitamin D levels three to four weeks after vaccination had a negative correlation with a decline in anti-SARS-CoV-2 IgG antibodies titer during six months after vaccination, and this finding was confirmed by multivariate binary logistic regression analysis. This means that vitamin D contributes to the stability of anti-SARS-CoV-2 antibody levels. Cecur F. and colleagues also identified that in [60]. Conversely, Chillon T.S. and colleagues concluded that the decrease in SARS-CoV-2 IgG concentrations over time was not associated with the 25(OH)D status [53]. However, that study was conducted in healthy subjects, which may have accounted for this difference. Also, after analyzing the data from the literature, it can be observed that the different age of patients might be the reason for such controversial results. We did not find statistically significant relations between vitamin D and levels of CD4+ and CD8+ cells. Still, our findings of correlation with anti-SARS-CoV-2 IgG antibodies titer lead to considering vitamin D’s importance in the formation and maintenance of humoral immune response after anti-SARS-CoV-2 mRNA vaccination.

One of the possible limitations of our study was the relatively small sample size, but our findings were based on the results of the largest dialysis center in Lithuania and the Baltic States. The choice of healthcare workers as a healthy control group can constitute a limitation of the study because this group is particularly exposed to repeated contact with the virus and frequent immunization, which may affect the antibody titer. Nevertheless, this group was chosen as a control, because during the second wave of the pandemic, when our study was organized, the very first groups to be vaccinated against COVID-19 were healthcare workers and dialysis patients in our country. Another limitation of our study could be the heterogeneity of the study population, as the ratio of males to females in the healthy control group was reversed compared to the study group. However, according to clinical studies, gender is less likely to determine an immune response [61], and, in our study, there was no statistically significant relation between anti-SARS-CoV-2 antibody titer, level of CD4+ and CD8+ cells and gender in the dialysis patients group with a randomized matched number of men.

## 5. Conclusions

Based on our study results, it was found that hemodialysis patients had decreased amounts of CD4+ and CD8+ cells and lower levels of anti-SARS-CoV-2 antibodies compared to healthy controls. This suggests that chronic hemodialysis could lead to diminished cellular immunity and humoral immune response to the anti-SARS-CoV-2 mRNA vaccination and reduced effectiveness in safeguarding against COVID-19. In addition, comorbidity in cardiovascular diseases is associated with a lower level of specific anti-SARS-CoV-2 antibodies titer. Vitamin D may be essential in maintaining the stability of the anti-SARS-CoV-2 antibody titer produced in hemodialysis patients. In contrast, the duration of dialysis treatment could be one of the factors decreasing anti-SARS-CoV-2 antibody titer and determining lower levels of CD4+ cells, thus, the worse cellular immune response.

## Figures and Tables

**Table 1:** Demographic and clinical characteristics of dialysis group.

Characteristic	Results (*n* = 45)	Normal Laboratory Ranges/ Recommendations, If Available
Age, year	62.7 ± 12.9	NA
Gender, male/female (%)	32 (71.1%):13 (28.9%)	NA
Comorbidities, n (%)		
Diabetes	11 (24.4)	NA
Cardiovascular diseases	32 (71.1)	NA
Malignancy	5 (11.1)	NA
Dialysis vintage, median [25–75%], months	44.4 [22.7–67.9]	NA
Dialysis access, arteriovenous fistula, n (%)	31 (68.9)	NA
spKt/V	1.38 ± 0.2	1.4 with a minimum delivered of 1.2 [16]
Hemoglobin, g/L	114.6 ± 13.2	100–120 [17]
Albumin, g/L	35.7 ± 3.0	35–52/ ≥40 [15]
C-reactive protein, mg/L	7.0 ± 4.8	<5
Phosphorus, mmol/L	1.6 ± 0.4	0.81–1.45/ to lower toward the normal range [18]
Calcium, mmol/L	2.28 ± 0.22	2.2–2.65/ to avoid hypercalcemia [18]
Parathormone, pmol/L	59.9 ± 43.9	1.26–6.7/ to maintain in the range of approximately 2 to 9 times the upper normal limit for the assay [18]
25-OH vitamin D level, nmol/L	55.7 ± 25.7	70–250
Cause of end-stage kidney disease, n (%)		NA
Chronic glomerulonephritis	7 (15.6)	
Hypertensive nephropathy	14 (31.1)	
Diabetes	8 (17.8)	
Chronic pyelonephritis	2 (4.4)	
Polycystic kidney disease	5 (11.1)	
Others	9 (20)	

Values for continuous variables are presented as mean ± standard deviation or Median [Q1–Q3], as appropriate; for categorical variables, as count (percentage). NA—not applicable.

**Table 2:** Comparison of lymphocyte subpopulations in study groups after full anti-SARS-CoV-2 vaccination.

Lymphocyte Subpopulations	Dialysis Patients Group (*n* = 45)	Healthy Control Group (*n* = 48)	*p*
CD3+ cells (×10^9^/L)	0.85 ± 0.39	1.39 ± 0.46	<0.001
CD4+ cells (×10^9^/L)	0.52 ± 0.26	0.82 ± 0.28	<0.001
CD8+ cells (×10^9^/L)	0.31 ± 0.19	0.53 ± 0.26	<0.001
CD19+ cells (×10^9^/L)	0.09 ± 0.06	0.22 ± 0.1	<0.001
CD16+/56+ cells (×10^9^/L)	0.26 ± 0.14	0.4 ± 0.23	0.002
CD4/CD8 ratio	2.1 ± 1.6	1.87 ± 1.0	0.769

**Table 3:** Associations between comorbidities and titer of anti-SARS-CoV-2 antibodies and levels of T cells in dialysis patients.

Presence of Comorbidities	Anti-SARS-CoV-2 Antibodies Titer (BAU/mL ± SD)	Level of CD4+ Cells (×10^9^/L)	Level of CD8+ Cells (×10^9^/L)
Subjects with diabetes	796.41 ± 719.17	0.57 ± 0.33	0.24 ± 0.16
Subjects without diabetes	1077.2 ± 1082.39	0.5 ± 0.24	0.33 ± 0.2
*p*	0.518	0.515	0.29
Subjects with cardiovascular diseases	830.89 ± 936.06	0.49 ± 0.24	0.29 ± 0.18
Subjects without cardiovascular diseases	1445.91 ± 1073.55	0.58 ± 0.31	0.34 ± 0.21
*p*	0.039	0.425	0.571
Subjects with malignancy	513.98 ± 387.08	0.41 ± 0.22	0.33 ± 0.17
Subjects without malignancy	1070.39 ± 1044.08	0.53 ± 0.26	0.3 ± 0.2
*p*	0.271	0.542	0.542

**Table 4:** Associations between clinical characteristics of dialysis patients and titer of anti-SARS-CoV-2 antibodies, level of CD4+, CD8+ cells.

Characteristic	Anti-SARS-CoV-2 Antibodies Titer (BAU/mL)		Level of CD4+ Cells (×10^9^/L)		Level of CD8+ Cells (×10^9^/L)	
	Spearman’s Correlation Coefficient	*p*	Spearman’s Correlation Coefficient	*p*	Spearman’s Correlation Coefficient	*p*
Dialysis vintage	0.251	0.118	−0.455	0.004	−0.307	0.06
spKt/V	0.171	0.297	−0.136	0.415	−0.082	0.624
Hemoglobin	0.028	0.865	0.123	0.426	0.097	0.53
Albumin	−0.107	0.487	−0.044	0.778	−0.271	0.076
C-reactive protein	−0.191	0.215	−0.106	0.495	0.001	0.996
Phosphorus	0.072	0.64	−0.003	0.986	−0.045	0.77
Calcium	0.172	0.263	0.018	0.91	0.047	0.761
Parathormone	0.016	0.92	0.185	0.23	0.13	0.401
25-OH vitamin D level	−0.378	0.019	−0.245	0.138	0.053	0.75
IgG, g/L	0.047	0.761	−0.088	0.568	0.094	0.545
IgM, g/L	0.139	0.369	−0.175	0.257	0.083	0.592
IgA, g/L	−0.027	0.861	0.075	0.631	−0.136	0.377
Dialysis vascular access *	NA *	0.441	NA *	0.943	NA *	0.591
Cause of end-stage kidney disease *	NA *	0.534	NA *	0.782	NA *	0.588

NA—not applicable; * Association was evaluated using the Mann–Whitney U or Kruskal–Wallis tests.

**Table 5:** Comparison of dialysis vintage and vitamin D level in the dialysis patients group according to decline in anti-SARS-CoV-2 titer and multivariate binary logistic regression analysis for evaluation of factors relevant to higher decline in anti-SARS-CoV-2 titer (>690 BAU/mL) during six months after vaccination.

Variable	Δ Anti-SARS-CoV-2 Titer <690 BAU/mL	Δ Anti-SARS-CoV-2 Titer >690 BAU/mL	*p*	Odds Ratio (95% CI), *p* Value
	Median [25–75%]			
Dialysis Vintage, Months	28.7 [18.2–57.5]	56.9 [24.1–91.1]	0.03	1.0388 (1.006–1.072), 0.021
Vitamin D level three–four weeks after second dose of anti-SARS-CoV-2 mRNA vaccine, nmol/L	69.0 [57.5–72.3]	53.9 [39.2–67.6]	0.04	0.971 (0.948–0.994), 0.016

*p* value by the Mann–Whitney U test; CI—Confidence Interval.

## Data Availability

Data are contained within the article and Supplementary Materials.

## Supplementary Materials

The following supporting information can be downloaded at: https://www.mdpi.com/article/10.3390/microorganisms12050861/s1, Figure S1. Relation between CD19+ cells and anti-SARS-CoV-2 titer in the blood of the healthy control group; Figure S2. Relation between CD4+ cells and anti-SARS-CoV-2 titer in the blood of dialysis patients; Figure S3. Distribution of responders to anti-SARS-CoV-2 mRNA vaccine in the dialysis patients group during the six-month follow-up period.
