# Supplementary figures and images for: Immune Response after Anti-SARS-CoV-2 mRNA Vaccination in Relation to Cellular Immunity, Vitamin D and Comorbidities in Hemodialysis Patients
Source: Microorganisms. 2024 Apr 25;12(5):861. doi: 10.3390/microorganisms12050861 (PMC11123711; doi:10.3390/microorganisms12050861)

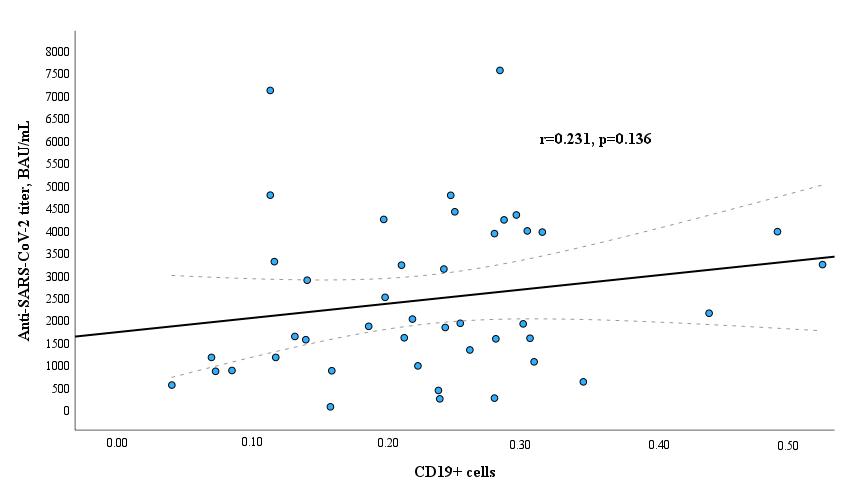

Supplement: Supplementary file 1 [file microorganisms-12-00861-s001.zip › microorganisms-2935108-supplementary/Supplementary Materials/Figure S1.jpg]

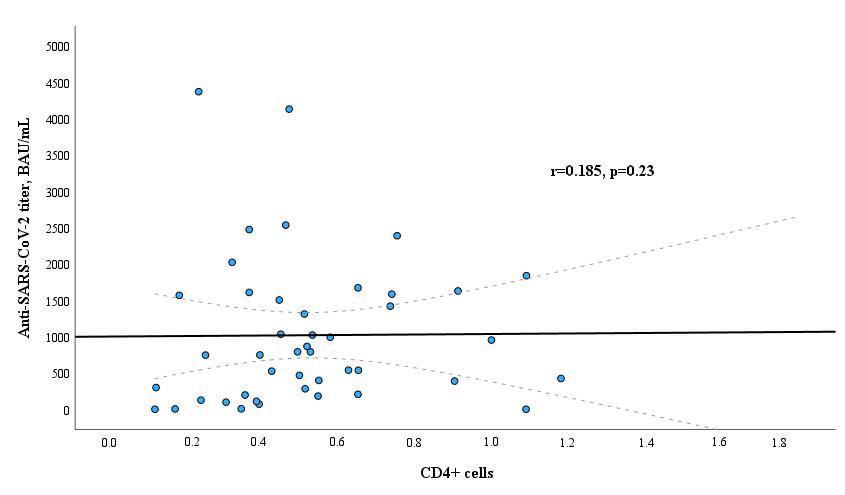

Supplement: Supplementary file 1 [file microorganisms-12-00861-s001.zip › microorganisms-2935108-supplementary/Supplementary Materials/Figure S2.jpg]

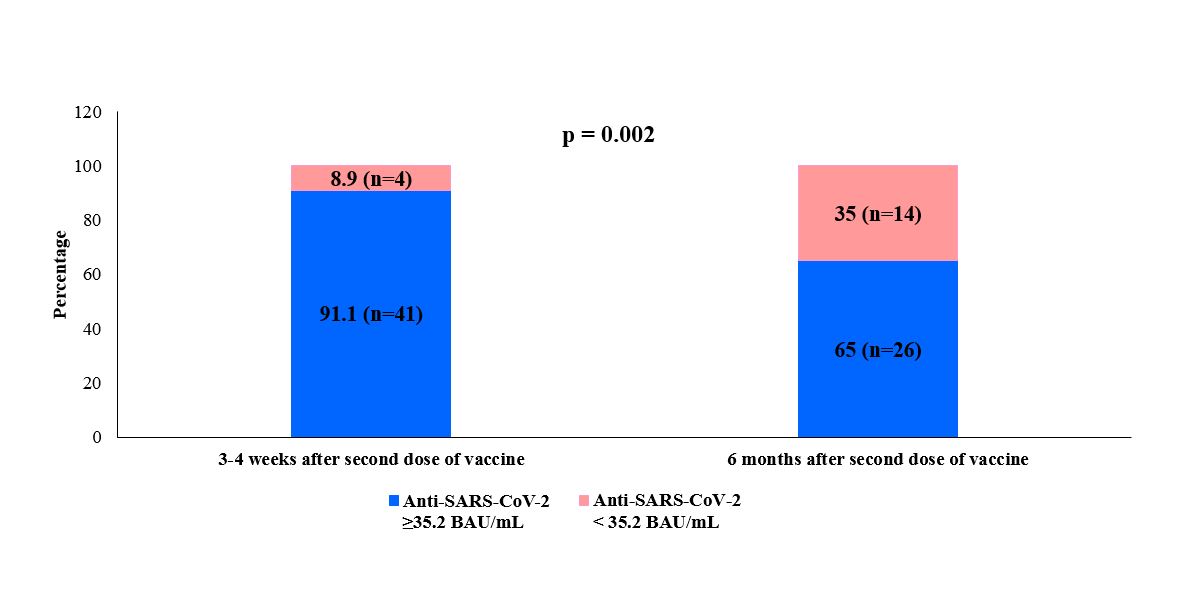

Supplement: Supplementary file 1 [file microorganisms-12-00861-s001.zip › microorganisms-2935108-supplementary/Supplementary Materials/Figure S3 .jpg]
